# Open access publishing – a quiet revolution

**DOI:** 10.1192/pb.bp.114.046813

**Published:** 2014-02

**Authors:** Jonathan Pimm

**Affiliations:** 1 The Editor, *Psychiatric Bulletin*

## Abstract

Radical changes are taking place in scientific publishing, driven by mandates from major research funders both in the UK and elsewhere. The publishing landscape is changing, and open access is increasingly being seen as a viable alternative to subscription-based business models. Although many issues are yet unresolved, even the large commercial publishers are developing stables of open access journals. To reach a wider audience, and to increase appeal to potential contributors deciding where to publish, the *Bulletin* has now become an open access journal with effect from this issue.

Revolutions tend to be chaotic and disturbing affairs. And yet the radical changes taking place in scientific publishing which threaten to rock the very foundations of the industry have been quiet and covert. The publishing revolutionaries aim to make all publicly funded research – and possibly all research – freely available to any curious reader. This is in contrast to the current conventional publishing model in which researchers use grant money to conduct studies, which are then published in an academic journal that is funded by journal subscriptions. The radical change, which has been discussed in previously smoke-filled rooms in universities and publishing houses alike for at least the past 10 years, is being driven by weighty institutions, including the British government-funded Research Councils UK (RCUK). Publishers now have a deadline requiring them to make all papers arising from projects supported by RCUK to be freely available (or open access) within a set period – 6 months for most biomedical research. The White House Office of Science and Technology Policy is following suit, as is the European Union.

Only occasionally does the matter enter the consciousness of those outside the arena, as it did for example at the end of last year, when a recent Nobel Prize winner called for academics to stop submitting their work to the pukka journals such as *Cell, Nature* and *Science*.^1^ Dr Randy Schekman, who runs a laboratory at the University of California, called for the boycott because he believes researchers and scientists are being inappropriately influenced by the need to get their work disseminated by these prestigious publications. He also claimed that the top-flight journals, aware of their prestigious position, artificially restrict the number of papers they accept.

At first sight the change to so-called open access might not seem so revolutionary; surely scientific research should be freely available to all? What really is the big deal? The answer, in part at least, is vast sums. Elsevier, the world's largest academic journal publisher – producing more than 90 journals including *The Lancet* as well as several others aimed at psychiatrists and allied professionals (e.g. *Schizophrenia Research, Biological Psychiatry* and *Psychiatric Research*) – in 2012 had a margin of 38% on revenues of £2.1 billion.^2^ Similarly, in 2011, German-owned Springer, which acquired BioMed Central in 2008, made 36% on sales of €875 million.^2^

Despite the profits, the publishers have historically argued that they incur significant costs to create and distribute a high-quality product. The matter is certainly not straightforward if alternative models of disseminating scientific knowledge are considered. Further, as with many revolutions, the battleground is littered with unseen incendiaries.

Clearly, publishing research findings is not cheap and, as with most things, someone has got to pick up the tab somewhere along the process. Previously it was the reader who paid, either individually or through organisation subscriptions. Current options for funding include the researcher or his/her affiliated institution. Such a situation is not without its problems nor its critics.^3^

Further, it is important to appreciate that the publication landscape has changed radically over the past decade or so. The San Francisco-based Public Library of Science (PLOS) was among the first group of organisations where publishing costs are met by charging authors a fee on acceptance. The group now has a stable of seven journals including *PLoS One, PLoS Biology* and *PLoS Medicine*. The organisation, which is non-profit, was backed by the Gordon and Betty Moore Foundation and received a $9 million grant to start up in 2002.^4^

Some commercial publishers have also been producing open access journals. For example, one of the largest, BioMed Central was founded in 2000 and now boasts dozens of journals, including many specialising in psychiatry and related disciplines: examples include *BMC Psychiatry, BMC Psychology* and *BioPsychoSocial Medicine*. Nature Publishing Group also produces a number of open access journals, while many mainstream publications offer open access on articles for a fee.

In addition to charging the author, other open access funding models have emerged. For example, PeerJ (https://peerj.com/) charges a one-off membership fee and authors also agree to act as reviewers. Other journals, for example *eLife* (http://www.elifesciences.org/) set up as a collaborative by several charitable foundations, are online-only and have no publication charges currently – although this situation may change. It should also be noted that Dr Schekman is an editor-in-chief at *eLife* and as such may not be entirely disinterested in calling for the boycott of rival publications.

Other key players in the world of academic publishing are the so-called learned societies – including the Royal College of Psychiatrists – which among other activities, produce their own journals such as this one, funded by monies from a variety of sources but mainly subscriptions from members and academic institutions. In recent years, many learned societies have faced falling journal sales resulting in declining revenues.

Those opposed to open access raise many concerns about the move including worries over the quality of articles. With the number of scientific journals seeming to grow exponentially, particularly those being entirely web-based, the question is how does one sort out the sheep from the goats?^3^

Predicting how the final publishing landscape will look in the future is difficult. The situation is still volatile. The proliferation undoubtedly provides researchers, academics and scientists more diverse opportunities to publish their work. Whether these new publications are viable in the longer term remains to be seen.

Here at the *Psychiatric Bulletin* there is a belief in the need for standards and the pursuit of excellence with the aim of betterment. And over the past 12 months, efforts have been made to improve things; the standard for article acceptance has been gradually increased and the review process has become more streamlined.

Further, to reach a wider audience and to increase appeal to potential contributors deciding where to publish, the *Bulletin* is now open access. Further still, publishing ahead of print has been introduced. Other strategies for quality improvement will include the production of themed editions focusing on hot topics such as culturally informed psychiatry, sustainability and public mental health.

And finally, to give a clear signal that the journal is eager to attract articles from health professionals, carers, lawyers, all members of the multidisciplinary team and all stakeholders involved in the treatment and care of people with mental illness, including, of course, patients themselves, there will be no cost to authors for at least the next 12 months and possibly longer.

## References

[1] Sample I Nobel winner declares boycott of top science journals. Guardian 2013; 9 12

[2] Economist Academic publishing: free-for-all. Open-access scientific publishing is gaining ground. Economist 2013; 4 5

[3] Wakeford R Open access publication – always a good thing? J Radiol Prot 2013; 33: E9–11 23914367 10.1088/0952-4746/33/2/e9

[4] Public Library of Science to launch new free-access biomedical journals with $9 million grant from the Gordon and Betty Moore Foundation. Gordon and Betty Moore Foundation 2002; 17 12 (http://www.moore.org/newsroom/press-releases/2002/12/17/public-library-of-science-to-launch-new-free-access-biomedical-journals-with-$9-million-grant-from-the-gordon-and-betty-moore-foundation).

